# Histological Changes Observed in Placentas Exposed to Medication-Assisted Treatment

**DOI:** 10.1155/2021/2175026

**Published:** 2021-10-07

**Authors:** Cara Staszewski, Kimberly M. Herrera, Elizabeth Kertowidjojo, Victoria Ly, Nicole Iovino, Diana Garretto, Cynthia Kaplan, Malini D. Persad, David J. Garry

**Affiliations:** ^1^Department of Obstetrics, Gynecology and Reproductive Medicine, Renaissance School of Medicine at Stony Brook University, Stony Brook, NY, USA; ^2^Department of Pathology, Renaissance School of Medicine at Stony Brook University, Stony Brook, NY, USA

## Abstract

**Introduction:**

To compare the effects of medication-assisted treatment on the placenta in pregnant women with opioid use disorder and uncomplicated pregnancies.

**Methods:**

This is a case-controlled study of pregnant women utilizing medication-assisted treatment, buprenorphine or methadone, which were matched to healthy uncomplicated controls by gestational age. Placental evaluations and neonatal outcomes were evaluated. Data analysis performed standard statistics and relative risk analysis with a *p* < 0.05 considered significant.

**Results:**

There were 143 women who met the inclusion criteria: 103 utilizing MAT, 41 buprenorphine and 62 methadone, and 40 uncomplicated matched healthy controls. The incidence of delayed villous maturation was 36% in the medication-assisted group compared with 10% in controls (RR 3.6: 95% CI 1.37-9.43; *p* < 0.01). The placental weight was greater (541 ± 117 g versus 491 ± 117 g; *p* = 0.02), and the fetoplacental weight ratio was lower (5.70 ± 1.1 versus 7.13 ± 1.4; *p* < 0.01) in the medication-exposed pregnancies compared with controls. The mean birth weight of the MAT newborns was significantly lower than that of the healthy controls (3018 ± 536 g versus 3380 ± 492 g; *p* < 0.01). When evaluating the subgroups of the MAT newborns, the birth weight of the methadone-exposed newborns (2886 ± 514 g) was significantly lower than that of the buprenorphine-exposed newborns (3218 ± 512 g; *p* < 0.01).

**Conclusion:**

Medication-exposed pregnancies have a greater incidence of delayed villous maturation, a larger placental size, and a decreased fetoplacental weight ratio compared to the healthy controls. Larger long-term follow-up studies to evaluate outcomes with the presence of delayed villous maturation are needed.

## 1. Introduction

The prevalence of opioid use disorder (OUD) has increased to nearly 7 per 1000 deliveries in the United States [1]. OUD during pregnancy has been associated with fetal growth restriction, placental abruption, fetal death, preterm labor, and intrauterine passage of meconium. Additionally, neonates are at risk for neonatal abstinence syndrome (NAS) [2]. Multimodal treatment of OUD includes addiction counseling, social supportive services, and pharmacologic treatment with medication-assisted treatment (MAT). Methadone, the traditional MAT over thirty years, and buprenorphine, a more recent medication, are used to control illicit opioid misuse and reduce opioid overdose [3].

Opioids cross the placenta, and the fetus is exposed. MAT dosing of the mother results in a lower fetal circulating level, approximately 10% of the maternal level [4]. Little is known about the placental changes, specifically histologic alterations, which occur in placental development associated with gestational MAT use.

We hypothesize that MAT in maternal opioid use disorder will have some effect on placental histology. Buprenorphine use for MAT has been described to have a more favorable newborn outcome compared to methadone [5]. We evaluated the effects of buprenorphine and methadone on the placental histological findings compared to normal uncomplicated controls.

## 2. Materials and Methods

This was an IRB-approved (CORHIS# 1064492) case-controlled study including women delivering between January 2007 and December 2017. Cases were identified using ICD codes for opioid use disorder, substance use disorder, and pregnancy. Identified women were divided into groups based on the type of MAT, buprenorphine (BUP) or methadone (METH). Uncomplicated healthy controls were defined as no MAT use and no maternal medical complications and were matched to study cases by gestational age at delivery. Women were excluded if they had gestational diabetes, maternal hypertension, preeclampsia, a multiple gestation, newborn growth restriction (<10^th^ percentile birth weight at delivery), clinical chorioamnionitis, and a positive urine toxicology results for substances other than methadone or buprenorphine and if they are without microscopic placental evaluation.

**Figure 1:**
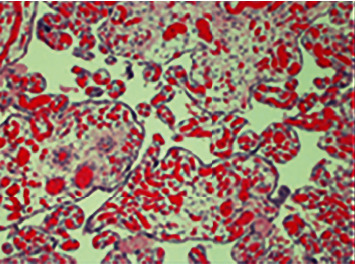
Placental dysmaturity. Photomicrograph of full-term placenta at 100x amplification showing placenta dysmaturity, characterized by large, irregularly shaped villi, decreased syncytial knots, hypervascularity, and villous edema.

All women meeting inclusion criteria had maternal, neonatal, and placental outcomes extracted from the medical record. Maternal outcomes included age, body mass index (BMI), race, gravidity, and any comorbid conditions. Neonatal outcomes included gestational age at delivery, birth weight, Apgar scores, need for morphine/NAS administration, length of stay in the hospital, respiratory distress (RDS), hyperbilirubinemia, and presence of newborn seizures. Placental outcomes included histological findings such as delayed villous maturation and placental weight. A devoted perinatal pathology team (EK and CK) read all histological slides. As part of the routine evaluation of the placentas, they were evaluated for the entire range of placental pathologies. Using the Amsterdam criteria for placental evaluation, placentas were examined fresh, and the umbilical cord, free membranes, and fetal and maternal surfaces are observed, and relevant measurements taken. Placentas are trimmed of excess membranes and the umbilical cord cut 2-3 cm from the fetal surface. Any soft clots are removed before weighing, and serial cross sections at 1-2 cm intervals are performed with any additional gross lesions recorded. A representative portion of the placenta with umbilical cord and membrane roll are fixed in 10% buffered formalin, and after one or more days of fixation, representative blocks are obtained [6]. The placenta was considered dysmature when histologic evaluation showed a degree of immaturity inappropriate for the gestational age. Delayed villous maturation was defined as a patchy to diffuse increase in the proportion of distal villi in a term or late preterm placenta with all or some of the following: increased villi diameter, increased stromal cellularity, nonperipheral capillaries, a thickened layer of villous trophoblast with uniformly distributed syncytiotrophoblastic nuclei, persistent cytotrophoblast, and a paucity of vasculosyncytial membranes [6, 7] (Figure 1). Placentomegaly was defined as a placental weight greater than 750 grams [8].

Sample size was calculated based on an approximate 10% incidence of placental dysmaturity and placentomegaly in controls and 35% in MAT-exposed placentas [9, 10]. Assuming a 2-sided 95% confidence interval with 80% power, with cases matched at 1 : 1 for each MAT medication to controls, we calculated a total sample size of 129, consisting of 43 MAT cases from buprenorphine, 43 cases from methadone, and 43 controls. Comparison of continuous data utilized Student's *t*-test or nonparametric alternatives with *p* value < 0.05 considered significant. Relative risk analysis was performed to assess the association of MAT with various binary outcome variables.

## 3. Results

Over the study period, there were 143 women meeting entry criteria with complete records and placental pathology results. There were 103 women utilizing MAT, 41 with buprenorphine and 62 with methadone, and 40 healthy uncomplicated controls. In comparing the MAT group with controls, the groups were similar in maternal age, BMI, and gestational age at delivery (Table 1). The MAT group was more likely to be Caucasian (89% versus 63%; *p* < 0.01) and more likely to have smoked (54% versus 17%; *p* < 0.01) compared with controls. The median gravidity for the MAT group was significantly greater than that for controls (gravidity 4, range 1-10 versus gravidity 2, range 1-5; *p* < 0.01). When comparing buprenorphine use with methadone use in the MAT group, they are similar for maternal age, gravidity, BMI, race, and gestational age at delivery. Methadone use had a lower median gestational age at delivery (median 39 weeks, range 34-41 weeks) compared to buprenorphine use (median 39 week, range 34-42 weeks; *p* = 0.03).

The incidence of delayed villous maturation was 36% for the MAT group, 34% with BUP and 35% with METH, and 10% in the controls (RR 3.6: 95% confidence interval 1.37-9.43; *p* < 0.01) (Table 2). When evaluating the BUP group with controls (RR 3.41: 95% confidence interval 1.23-9.49; *p* = 0.02) or the METH group with controls (RR 3.71: 95% confidence interval 1.39-9.93; *p* < 0.01), both MAT groups had significantly greater delayed villous maturation. When comparing BUP with METH, there was a similar incidence of delayed villous maturation (RR 0.92: 95% confidence interval 0.54-1.57; *p* = 0.76). The overall placental weight was greater in the MAT exposed women compared to controls (541 ± 117 g versus 491 ± 117 g; *p* = 0.02) but similar between the subgroups of MAT (*p* = 0.22) (Table 2). When evaluating the fetoplacental weight ratio, the MAT-exposed pregnancies had a significantly lower ratio (5.70 ± 1.1 versus 7.13 ± 1.4; *p* < 0.01) compared with controls. The fetoplacental weight ratio did not differ between the BUP and METH groups (*p* = 0.05) (Table 2).

The mean birth weight of the MAT newborns was significantly lower than that of the healthy controls (3018 ± 536 g versus 3380 ± 492 g; *p* < 0.01) (Table 1). When evaluating the subgroups of the MAT newborns, the mean birth weight of the METH newborns was significantly lower than that of the BUP exposed newborns (2886 ± 514 g versus 3218 ± 512 g; *p* < 0.01). The preterm < 37 weeks of delivery rate and the incidence of small for gestational age newborn were similar between groups. The incidence of respiratory distress was higher in the METH group compared with controls (31% versus 13%; *p* < 0.04). The incidence of neonatal abstinence syndrome treatment with morphine was similar between the METH group and BUP group (48% versus 32%; *p* = 0.09) with no treatment required in the control group. Newborn length of stay was great in the MAT group compared with controls (11.0 + 7.9 days versus 2.6 + 1.1 days; *p* < 0.001) (Table 1).

## 4. Discussion

The use of MAT in opioid use disorder during pregnancy results in a greater incidence of delayed villous maturation, a larger placental size, and a decreased fetoplacental weight ratio compared to the healthy unexposed controls. Like our findings, Serra et al. evaluated the presence of delayed villous maturation of placentas in pregnant women using MAT and found a correlation of a greater dysmaturity rate in opioid-exposed pregnancy compared to controls, specifically with methadone [9]. Our study found a similar incidence of placental dysmaturity when comparing buprenorphine and methadone exposure. We excluded other substance use disorders based on toxicology of the mother and newborn. The identification of placental dysmaturity can have a role in further understanding of newborn outcomes unexplained by other methods, including newborn immediate evaluation or umbilical cord pH assessment [11].

Opioid use is increasing among pregnant women. Understanding the risks associated with both short- and long-term uses of MAT during pregnancy continues to be an area of investigation. Maternal substance use disorder has various effects on the fetus including poor fetal growth, preterm birth, birth defects, and long-term cognitive development. The placenta, in addition to its role of supporting nutrient exchange for the fetus, serves as a window to the intrauterine environment. Delayed villous maturation represents a spectrum of placental disorders with decreased vascularization of the chorionic villi with a reduced number of syncitiocapillary membranes [9]. Delayed villous maturation occurred in 36% in women utilizing MAT; however, this could increase if women with underlying medical disorders were included [11]. Conversely, certain maternal conditions which affect uteroplacental blood flow, such as kidney disease, hypertension, or preeclampsia, can alter the transfer of MAT across the placenta and further effect of villous maturation. [4, 12]

The fetoplacental ratio in our women exposed to MAT demonstrated a reduced ratio, or larger placentas, when compared to controls. The use of fetal/placental weight ratios allows for correction of the size of the newborn and offers another means to assess placental size. A fetoplacental weight ratio of approximately 7 at term can be considered normal and was our findings in the control population [6, 8]. In a complete, trimmed placenta, finding a ratio outside this normal range for that gestational age should be considered for further histologic examination [8]. The common causes of unusually large placenta are maternal diabetes mellitus, severe maternal anemia, fetal anemia, congenital infections, large intervillous thrombi, and a large blood clot beneath the chorion of the placenta [13, 14]. It has been suggested that there is increased risk with high placental weight secondary to placental villous edema, which may alter gas exchange between the mother and fetus [15].

Limitations to our study include the retrospective style of the study, missing analysis, and the limited numbers, especially in the control group. In the MAT group, the timing of medication initiation in pregnancy and the severity of opioid use disorder could not be quantified and compared in a meaningful manner. As an institutional guideline, placentas from uncomplicated pregnancies are most often not sent to pathology for microscopic evaluation, thus significantly limiting the number of controls over the study period. The exclusion of those women with other substances on urine toxicology testing and those without microscopic evaluation of the placenta is an additional limitation for the study. The strengths of our study include the placental pathologists (EK and CK) with expert knowledge in opioid effects on the placenta, and the number of opioid-exposed pregnancies without concomitant medical disorders or fetal growth disorders allows for an adequate MAT group.

## 5. Conclusions

Women with opioid use exposure during pregnancy should have histologic placental pathology performed with potential identification of delayed villous maturation. Further investigation of the long-term outcomes of newborns with placental delayed villous maturation is needed to better understand the implications of this abnormality.

## Figures and Tables

**Table 1:** Maternal demographics and neonatal outcomes of MAT-exposed gestations compared with uncomplicated gestational age-matched controls.

	Buprenorphine *N* = 41	Methadone *N* = 62	MAT *N* = 103	Controls *N* = 40
Maternal				
Maternal age (years)	29.7 ± 4.6	29.6 ± 4.7	29.7 ± 4.7	29.4 ± 6.5
BMI (kg/m^2^)	29.6 ± 6.2	28.8 ± 6.1	29.1 ± 6.1	27.3 ± 5.8
Race				
Caucasian	38 (93)	54 (87)	92 (89)^∗^	25 (63)
Black	2 (5)	1 (2)	3 (3)	2 (5)
Hispanic	1 (2)	3 (5)	4 (4)^∗^	9 (23)
Other	0 (0)	1 (2)	1 (1)	3 (8)
Smoker	18 (44)	38 (61)	56 (54)^∗^	7 (17)
GA delivery (weeks)	39 (34-42)	39 (34-41)^‡^	39 (34-42)	39 (35-42)
Delivery < 37 weeks	4 (10)	10 (16)	14 (14)	3 (8)
Neonatal				
Birth weight (grams)	3218 ± 512	2886 ± 514^∗‡^	3018 ± 536^∗^	3380 ± 492
Small for gestational age	4 (10)	4 (6)	8 (8)	3 (8)
5-minute Apgar score	8.5 ± 1.4	8.6 ± 0.9	8.6 ± 1.1	8.8 ± 0.7
LOS hospital (days)	8.7 ± 5.1^∗^	12.6 ± 9.1^∗‡^	11.0 ± 7.9^∗^	2.6 ± 1.1
Required morphine	13 (32)^∗^	30 (48)^∗^	43 (42)^∗^	0 (0)
Respiratory distress	8 (20)	19 (31)^∗^	27 (26)	5 (13)

Categorical data presented as *N* (%) and continuous data presented as the mean ± SD or median (range). BMI: body mass index; GA: gestational age; MAT: medication-assisted treatment; LOS: length of stay. ^∗^*p* < 0.05 compared with the control group. ^‡^*p* < 0.05 buprenorphine group compared the methadone group.

**Table 2:** Placental findings in pregnant women using medication-assisted treatment (MAT) for control of opioid use disorder compared with healthy gestational age-matched control.

	Buprenorphine *N* = 41	Methadone *N* = 65	MAT *N* = 103	Controls *N* = 40
Placental dysmaturity	14 (34)	23 (35)	37 (36)	4 (10)^∗∗^
Placenta weight (grams)	559 ± 118	530 ± 116	541 ± 117	491 ± 117^∗∗^
Placental weight > 750 grams	3 (7.3)	4 (6.2)	7 (6.8)	1 (2.5)
Fetoplacental weight ratio	5.95 ± 1.3	5.54 ± 0.79	5.70 ± 1.1	7.13 ± 1.4^∗∗^

Categorical data presented as *N* (%) and continuous data presented as the mean ± SD. MAT: medication-assisted treatment. ^∗∗^*p* < 0.05 MAT groups compared to controls. ^‡^*p* < 0.05 buprenorphine group compared to methadone group.

## Data Availability

The data used to support the findings of this study are available from the corresponding author upon request.
